# A Few Prominent Types of Heart Disease

**Published:** 1915-06

**Authors:** W. T. Shell

**Affiliations:** Corsicana, Texas


THE
### TEXAS MEDICAL JOURNAL
Dr. F. E. Daniel, Founder. Established July, 1885
MRS. F. E. DANIEL, - Publisher and Managing Editor
Published Monthly.—Subscription, $1.00 a Year.
VOL. XXX AUSTIN, JUNE, 1915 No. 12
The publisher is not responsible for views of contributors

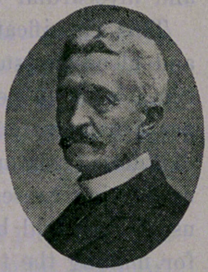

### Original Articles.

### A Few Prominent Types of Heart Disease.

BY DR. W. T. SHELL, CORSICANA, TEXAS.

For the past few months I have been especially interested in the study of diseases of the heart, and have come to realize more forcibly than ever before how important and direct a bearing this subject has on a large per cent of the cases which come under my observation. In fact, I find that in many instances heart disturbances, which might readily be pronounced effect, are in reality the very root of the evil. Hence, I wish to emphasize the thought that it may behoove us all to lay more stress upon heart disorders as prime factors in a great number of ailments which we are called on to combat.

To touch, even lightly, on all the salient points of so vast a subject would require far more time and study than I have been able to devote to this topic. I have only undertaken a brief summary of a few important types.

Dr. Richard Cabot of Boston classifies the four common types of heart disease as follows, the frequency of each in the order named: Rheumatic, syphilitic, arteriosclerotic and nephritic. Under the head of rheumatic is weakened heart and valvular disease, with or following rheumatic fever. Chorea and acute tonsilitis in young persons. Under syphilitic are syphilitic aortitis and aneurism, with positive Wassermann reaction. Under arteriosclerotic are weakened heart in elderly persons, angina pectoris, etc., with a negative Wassermann. Under nephritic are the cases of weakened and enlarged heart, with marked urinary abnormalities, Bright's disease, etc. Then follows goiter heart, obese heart and myocardial weakness.

This classification was made possible by Wassermann reaction and blood pressure measurements as a routine. In the first four types were 93 per cent of all cases of heart disease; the other 7 per cent were goiter heart and doubtful cases. For purposes of diagnosis, treatment and prognosis, it is necessary to know the underlying causes. The Wassermann is not observed by some of us as it should be, for the reason that we are not properly fitted for making the test. However, we can collect the blood and send it to the laboratories of larger cities where the test will be made for a reasonable sum, say, $5.00. The average charge for collecting blood and making test being, I believe, $25.

The sphygmomanometer is of prime importance. It should be and is being used as a routine more and more by the general practitioner. By this means a diagnosis of kidney disease can be made, when a urinalysis will fail, and is of the greatest importance in latent nephritis.

Mitral stenosis is the lesion of most importance produced by the rheumatic condition. It is a disease peculiar to young girls. Some of the most latent symptoms have the loudest murmurs and are discovered accidentally. Mitral stenosis is the one cause of embolism. Left hemiplegia is a common complication, but the prognosis is good.

After compensation has been established there is no need of keeping the patient quiet. When a person suffering from mitral stenosis has reached the age of twenty-one years there is very little danger of the heart trouble proving fatal, in many instances they live to an old age.

There are four types of irregularity of the heart:

1. Absolute perpetual arythmia.

2. Premature contraction or extra systole which skip every third or fourth beat, and is not a bad sign.

3. Senile arythmia.

4. Heart block, while not usually of great importance, may sometimes be so severe the patient will have convulsions. When in doubt, have patient walk up and down room three or four times, and it will be more irregular if of any seriousness; if not, it will be made better.

Aneurism of the aorta is only due to one cause, syphilis. In the manifestation of the three groups of symptoms, pain is very prominent and is more apt to be in the shoulder, though it may be anywhere in the chest, suggesting angina, but lasting a longer period.

Of the pressure symptoms, there may be a pressure pain of the descending aorta. There is usually a change, a loss of, or a diminution in the voice, and with that the cough, making a loud and peculiar noise but bringing up nothing. This cough has the characteristics of obstruction. The pressure may be pronounced enough to affect the pulse and possibly the pupils.

There is also the tracheal tug from pressure. The physical signs are bulging and pulsation. Generally there is an audible murmur. The percussion sounds are dull. In many cases the diagnosis would be impossible without the X-ray. X-ray is not necessary, however, in the diagnosis of most troubles in the chest.

Abdominal aneurism is extraordinary and among the great rarities. Patients sometimes come to us very much frightened with a decided pulsation in the abdomen. We can usually assure them that it is the abdominal aorta, as it can be right under the skin. A dynamic aneurism produces pain in front over the aorta. True aneurismal pain is in shoulder and back. In aneurism Wassermann is usually positive. In aortic disease of middle life without rheumatism, always look for syphilis, with generally a large heart apex in sixth or seventh interspace.

Arteriosclerosis raises blood pressure. We have a hypertension when the heart is enlarged and a hypotension when it is small. Temporary causes of high blood pressure are pain, apoplexy, hemorrhage, meningitis. An elevation of pressure in pregnancy is an indication of eclampsia. A low pressure is not of so much importance, but is found in tuberculosis, anemia, surgical shock, general peritonitis. The systolic reading is necessary in every case we see, the diastolic not being considered of much importance.

Arteriosclerosis is the commonest of all causes of sudden death. One of the most frequent symptoms in old people is attacks of vertigo. These same patients will generally later have apoplexy. Temporary attacks of fainting or so-called convulsion, paresthesia and mental lapses in old people are due to coronary sclerosis.

Acute indigestion is never the cause of sudden death—newspaper reports to the contrary notwithstanding. Deaths which occur after a hearty meal are invariably attributed (by reporters) to acute indigestion. In reality they are caused from plugging of the coronary artery, which condition is brought about by too great a strain placed upon the heart, through overloading the stomach.

There are three causes of sudden death, viz: Plugging of the coronary artery, pancreatitis and fulminating appendicitis.

Angina pectoris is one of the effects of coronary sclerosis. A pain in the arm, which is produced by emotion or exercise, and ceases by rest, is angina. The pain of angina pectoris is generally of brief duration, usually being a matter of minutes or seconds, not hours. Many cases of mild or varying degrees of angina pectoris are constantly mistaken for indigestion, because there is such a tendency to gas formation in the stomach. In chronic interstitial nephritis there is an enlarged and weakened heart with a high blood pressure. The right side of the heart may fail from a granular kidney, and other high blood pressure conditions.

Absolute rest for an indefinite time is often prescribed inadvisedly in cases of mitral stenosis, continued idleness being fatal to the well being of any individual. Of course it is necessary to discriminate most carefully in advising work for such patients, but systematic and congenial employment is the very keynote to happiness, which is, perhaps, the doctor's most valuable ally.

*Read before the Navarro County Medical Society, December 7, 1914.

